# Toxicokinetics of recombinant human fibroblast growth factor 21 for injection in cynomolgus monkey for 3 months

**DOI:** 10.3389/fphar.2023.1176136

**Published:** 2023-05-23

**Authors:** Chao Lu, Lei Jin, Jianing Bi, Hongyi Jin, Xinyi You, Lulu Peng, Haibing Fan, Huan Wang, Liangshun Wang, Zhengkai Fan, Xiaojie Wang, Baohua Liu

**Affiliations:** ^1^ Department of Neurological Rehabilitation, The Second Affiliated Hospital of Wenzhou Medical University, Wenzhou, China; ^2^ School of Pharmacological Sciences, Wenzhou Medical University, Chashan University Park, Wenzhou, China; ^3^ Laboratory of Zhejiang Province for Pharmaceutical Engineering and Development of Growth Factors, Collaborative Biomedical Innovation Center of Wenzhou, Wenzhou, China; ^4^ Research Units of Clinical Translation of Cell Growth Factors and Diseases, Chinese Academy of Medical Science, Wenzhou, China

**Keywords:** recombinant human fibroblast growth factor 21, enzyme-linked immunosorbent assay, toxicokinetics, anatomy, pathology

## Abstract

**Introduction:** Recombinant human fibroblast growth factor 21 (FGF-21) is a potential therapeutic agent for multiple metabolic diseases. However, little is known about the toxicokinetic characteristics of FGF-21.

**Methods:** In the present study, we investigated the toxicokinetics of FGF-21 delivered via subcutaneous injection *in vivo*. Twenty cynomolgus monkeys were injected subcutaneously with different doses of FGF-21 for 86 days. Serum samples were collected at eight different time points (0, 0.5, 1.5, 3, 5, 8, 12, and 24 h) on day 1, 37 and 86 for toxicokinetic analysis. The serum concentrations of FGF-21 were measured using a double sandwich Enzyme-linked immunosorbent assay. Blood samples were collected on day 0, 30, 65, and 87 for blood and blood biochemical tests. Necropsy and pathological analysis were performed on d87 and d116 (after recovery for 29 days).

**Results:** The average AUC_(0-24h)_ values of low-dose FGF-21 on d1, d37, and d86 were 5253, 25268, and 60445 μg h/L, and the average AUC_(0–24h)_ values of high-dose FGF-21 on d1, d37, and d86 were 19964, 78999, and 1952821 μg h/L, respectively. Analysis of the blood and blood biochemical indexes showed that prothrombin time and AST content in the high-dose FGF-21 group increased. However, no significant changes in other blood and blood biochemical indexes were observed. The anatomical and pathological results showed that continuous subcutaneous injection of FGF-21 for 86 days did not affect organ weight, the organ coefficient, and histopathology in cynomolgus monkeys.

**Discussion:** Our results have guiding significance for the preclinical research and clinical use of FGF-21.

## 1 Introduction

Fibroblast growth factor 21 (FGF-21) is an endocrine hormone capable of improving metabolic dysfunction and related complications by regulating the energy balance (Degirolamo et al., 2016; BonDurant and Potthoff, 2018; Geng et al., 2020). FGF-21 has been shown to significantly reduce blood sugar levels and body weight by regulating carbohydrate and fat homeostasis in diabetic or obese animal models (Kharitonenkov et al., 2007; Adams et al., 2013; Gaich et al., 2013; Markan and Potthoff, 2016; BonDurant et al., 2017; Kharitonenkov and DiMarchi, 2017; Stanislaus et al., 2017). In rodent models of diet-induced nonalcoholic hepatitis (NASH), FGF-21 deficiency exacerbates hepatic steatosis, inflammation, and fibrosis, while FGF-21 supplementation attenuates these pathological changes and reduces NASH markers level (Fisher et al., 2014; Tanaka et al., 2015). In a phase IIa trial of Pegbelfermin (a PEGylated fibroblast growth factor 21 analogue) treatment in NASH patients, a significant decrease in the plasma level of N-terminal type III collagen propeptide (PRO-C3), a hepatic fibrosis marker that reflects the severity and fibrosis stage, was observed (Sanyal et al., 2019). Liver biopsies confirmed that Pegbelfermin can significantly reduce the liver’s density fat fraction and plasma markers of cirrhosis and fibrosis (Sanyal et al., 2019). Efruxifermin, a long-acting Fc-FGF-21 fusion protein, was also shown to reduce liver density fat fraction and fibrosis in NASH patients (Harrison et al., 2021). Furthermore, FGF-21 attenuates atherosclerosis and inflammation in diet-induced obese ApoE-deficient rodents (Lin et al., 2015; Jia et al., 2018) and protects against cardiomyopathy and kidney disease in people with diabetes (Wu et al., 2017; Zhao et al., 2017).

FGF-21 is a therapeutic option for chronic metabolic diseases such as NASH and diabetes (Gaich et al., 2013; Talukdar et al., 2016; Geng et al., 2020; Zarei et al., 2020; Talukdar and Kharitonenkov, 2021). However, chronic metabolic diseases such as diabetes and NASH require long-term medication. Therefore, it is of great significance to understand the toxicokinetic characteristics of FGF-21 after long-term use. In the present study, the toxicokinetic characteristics of FGF-21 in cynomolgus monkeys were evaluated by a sandwich ELISA method. After subcutaneous injection of FGF-21, we collected the serum of cynomolgus monkeys at eight different time points on day 1 (d1), d37, and d86. The detected data were used for toxicokinetic analysis.

Our results show that the serum level of FGF-21 in cynomolgus monkeys was positively correlated with the concentration and frequency of subcutaneous injection. C_max_ increased with the increase in the dose of FGF-21. During the injection of FGF-21, t_1/2z_, T_max_, and CLz/F of FGF-21 fluctuated within a certain range and were not dose-related. Over time, t_1/2z_ tended to increase and CLz/F gradually decreased. The drug accumulation analysis confirmed that FGF-21 had accumulated in cynomolgus monkeys. Autopsy and pathological examination were carried out, and no abnormality was found.

## 2 Materials and methods

### 2.1 Reagents

Recombinant human FGF-21 for injection (batch number: 20150101) and its vehicle (10 mL/tube and 120 mL/tube, batch numbers 20150301 and 20150901, respectively) were produced and provided by Wenzhou Medical University Biological and Natural Medicine Development Center Co., Ltd. The human FGF-21 Immunoassay Kit was purchased from IMD Company (Product No.: 31180, Lot number: HFT012, Shenzhen, China).

### 2.2 Methods

#### 2.2.1 Method validation: accuracy and precision

##### 2.2.1.1 Linearity

A series of FGF-21 standard solutions was prepared with final concentrations of 4,000, 2000, 1,000, 500, 250, 125, and 62.5 pg/mL. The absorbance (A) was plotted against the drug concentration (C), and a sigmoid curve was drawn based on a four-parameter log-logistic (4-PL) model. The experiment was repeated 10 times to obtain the standard curve of the FGF-21 ELISA kit.

##### 2.2.1.2 Precision and accuracy

Five QC samples (4,000, 3,200, 800, 160, and 62.5 pg/mL) were used to analyze the precision and accuracy. The intra-batch precision and accuracy were determined by measuring each quality control sample five times on the same enzyme-labeled plate. Two wells were included for each QC sample, and the average value was taken. The inter-batch precision and accuracy were investigated by measuring the OD values of each quality control sample on different enzyme-labeled plates of six independent analysis batches. The ratio of the standard deviation to the mean value is the precision, expressed as RSD%. The ratio of the average value of the measured results to the theoretical value is the accuracy, expressed as RE%.

### 2.3 Animals, dosage, and injection of FGF-21

Twenty cynomolgus monkeys (SPF grade, 3–5 years old, 10 female and 10 males, males weighing 2.85–3.90 and females 2.75–3.50 kg) were purchased from Suzhou Xishan Zhongke Laboratory Animal Co., Ltd. (Suzhou, China). Monkeys were randomly divided into two groups (n = 10 animals per group). Low- and high-dose FGF-21 were subcutaneously injected for 86 days. After the last injection (d86), the animals were allowed to recover for 29 days. The animals were fed normally during the drug administration period.

The pharmacological doses of FGF-21 in the diabetic model ob/ob mice were 0.125, 0.375, and 1.125 mg/kg, respectively; all of which showed efficacy without significant dose-dependent relationships. According to the body surface area, the equivalent doses of FGF-21 for cynomolgus monkeys were 0.04, 0.11, and 0.34 mg/kg, respectively. Based on previous literature reports (Kharitonenkov et al., 2005; Kharitonenkov et al., 2007; Gaich et al., 2013), the low dose of cynomolgus monkeys after repeated administration for 3 months was set at 0.7 mg/kg, which is approximately 6 times the pharmacological equivalent dose (based on medium dose). The high dose was set at 2.8 mg/kg is 25 times the pharmacological equivalent dose.

### 2.4 Determination of serum FGF-21 levels

The serum concentration of FGF-21 was measured using the ELISA method. The OD value was measured in a 96-well plate using a microplate reader at a wavelength of 450 nm.

### 2.5 Blood collection and analysis

6 mL of blood was collected from the forelimb veins of animals during the adaptation stage d7 and d22 (the mean was denoted as d0); the drug administration stage d30, d65, d87; and the recovery stage rd29. 2 mL was subjected to EDTA-K2 anticoagulation for blood cell analysis while 1 mL was treated with sodium citrate anticoagulant and centrifuged to obtain plasma for coagulation function analysis. 3 mL was placed in a vacuum tube with separation gel/coagulant for 30 min and then centrifuged to acquire serum for biochemical and electrolyte analysis.

### 2.6 Hematological testing

EDTA-K2 anticoagulation-treated blood was analyzed for blood routine indexes using the ADVIA 2120 multi-species hematology analyzer within 4 h. Examined blood routine indexes include white blood cell count (WBC) (which included percentages of Neutrophil N%, Lymphocyte L%, Eosinophilic E%, Monocyte M%), red blood cell count (RBC), hemoglobin (HB), red blood cell volume (HCT), mean red blood cell volume (MCV), mean red blood cell hemoglobin (MCH), mean erythrocyte hemoglobin concentration (MCHC), platelet count (PLT), reticulocyte count (Ret %).

Sodium citrate-treated blood was used to determine the prothrombin time (PT) and partially activated prothrombin time (APTT) using the ACL9000 fully automatic coagulation instrument.

### 2.7 Blood biochemical testing

Part of the collected serum was used to measure the biochemical indexes using an automated biochemical analyzer. Detected biochemical indexes include alanine aminotransferase (ALT), aspartate aminotransferase (AST), alkaline phosphatase (ALP), serum urea nitrogen (BUN), creatinine (Crea), total protein (TP), albumin (ALB), blood glucose (GLU), total cholesterol (TCHO), total bilirubin (T-BIL), triglyceride (TG), creatine Phosphokinase (CK), gamma-glutamyltransferase (gamma-GT).

Serum concentrations of potassium (K+), sodium (Na+), and chlorine (Cl-) ions were measured by the EX-180 electrolyte analyzer.

### 2.8 Clinical observation and histopathology

Cynomolgus monkeys were weighed using an electronic scale (OCS-W-100 kg) three times per day on d0, d24, d57, and d87 after injecting FGF-21. Cynomolgus monkeys were also weighed at the end of the recovery period (rd29). After the administration period (d87, n = 6) and after the recovery period (rd29, n = 4), animals were dissected.

The brain, cerebellum, brain stem, pituitary gland, eyeball, heart, aorta, lung (main bronchus), trachea, salivary glands, liver, pancreas, gallbladder, kidney, spleen, thymus, spinal cord (neck, chest, waist), sciatic nerve, mammary gland, uterus, ovary, fallopian tube, vagina, cervix, prostate gland, testis, epididymis, seminal vesicle, adrenal gland, thyroid gland, parathyroid gland and distal lymph node, esophagus, stomach, duodenum, jejunum, ileum, cecum, colon, rectum, bladder, skin, skeletal muscle, the administration site, lymph nodes related to administration, bone marrow (sternum), and femur were resected. Finally, the adipose tissue and connective tissue were removed and blood and tissue fluid were absorbed with filter paper. HE staining was performed to analyze organ histopathology. The heart, liver, spleen, kidney, brain, adrenal gland, thymus, testis, epididymis, uterus, ovary, and thyroid (including parathyroid gland) were weighed. For each organ, the organ coefficient was calculated using the following formula:

$$Organ\;coefficient=\frac{organ\;weight\;\left(g\right)}{body\;weight\;\left(kg\right)}.$$

### 2.9 Toxicokinetic analysis

Serum was collected and FGF-21 concentrations were determined on d1, d37, and d87 at 0, 0.5, 1.5, 3, 5, 8, 12, and 24 h after injecting FGF-21. The plasma FGF-21 concentration-time relationship was analyzed using Drug and Statistics (DAS) 3.2.8 software (Beijing Bozhiyin Technology Co., Ltd.). The non-compartmental model was used to calculate the pharmacokinetic parameters, including the area under the drug–time curve (AUC(0-t)), t1/2z, Tmax, Cmax, and CLz/F. Except for individual data, all toxicokinetic parameters in each test group are expressed as mean ± standard deviation ( $\bar{x}\pm s$ ). Mapping and statistical analysis results were obtained by GraphPad Prism 8.

## 3 Results

### 3.1 Quantitative range of the ELISA kit

**FIGURE 1:**
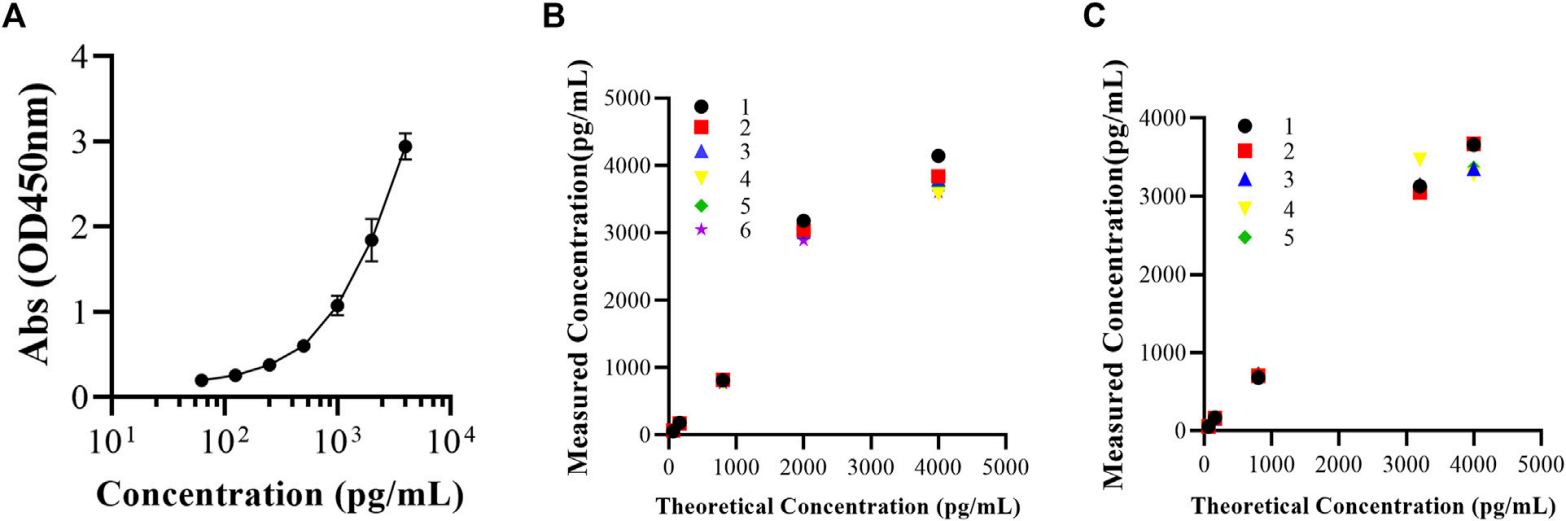
Validation of applicability of FGF-21 ELISA kit. **(A)** Mean standard curves of FGF-21 ELISA kit (Abs = 7.210–7.208/[1+(C/6085.0)1.063], R2 = 0.9998, $\bar{X}\pm s$ , n = 10). **(B,C)** The intra-batch (b, n = 6)/inter-batch (c, n = 5) precision and accuracy of FGF-21 were determined by ELISA.

The standard curve of the FGF-21 ELISA kit showed a quantitative relationship between FGF-21 concentration (C) and OD value (y). The equation is y = 7.210–7.208/[1 + (C/6085.0)1.063], and the correlation coefficient (r) is 0.9998. The quantitative range of the ELISA kit was from 62.5 pg/mL to 4,000 pg/mL (Figure 1A).

### 3.2 Precision and accuracy

As shown in Figure 1, in the concentration range of 62.5–4,000 pg/mL, the intra-batch and inter-batch RSD% values of FGF-21 were 2.9%–8.0% and 3.1%–12.2%, respectively. RE% values of the intra-batch and inter-batch of FGF-21 were −13.4% to 0.3% and −5.7% to 5.4%, respectively (Table 1). The RSD% and RE% values of QC samples are less than 20%, so the accuracy and precision of the ELISA kit are acceptable (Figures 1B,C).

**TABLE 1:** Precision and accuracy of FGF-21 determined by ELISA ( $\bar{X}\pm s$ ).

Concentration (pg/mL)	Intra batch (n = 5)			Inter batch (n = 6)		
	Measured value (pg/mL)	RSD (%)	RE (%)	Measured value (pg/mL)	RSD (%)	RE (%)
4,000	3,464.3 ± 186.2	5.4	−13.4	3,773.3 ± 210.5	5.6	−5.7
3,200	3,178.8 ± 165.5	5.2	−0.7	3,031.7 ± 92.6	3.1	−5.3
800	710.2 ± 20.3	2.9	−11.2	797.0 ± 24.6	3.1	−0.4
160	160.5 ± 8.8	5.5	0.3	168.7 ± 8.5	5.1	5.4
62.5	56.0 ± 4.5	8.0	−10.5	62.8 ± 7.6	12.2	0.4

### 3.3 Toxicokinetic analysis

**FIGURE 2:**
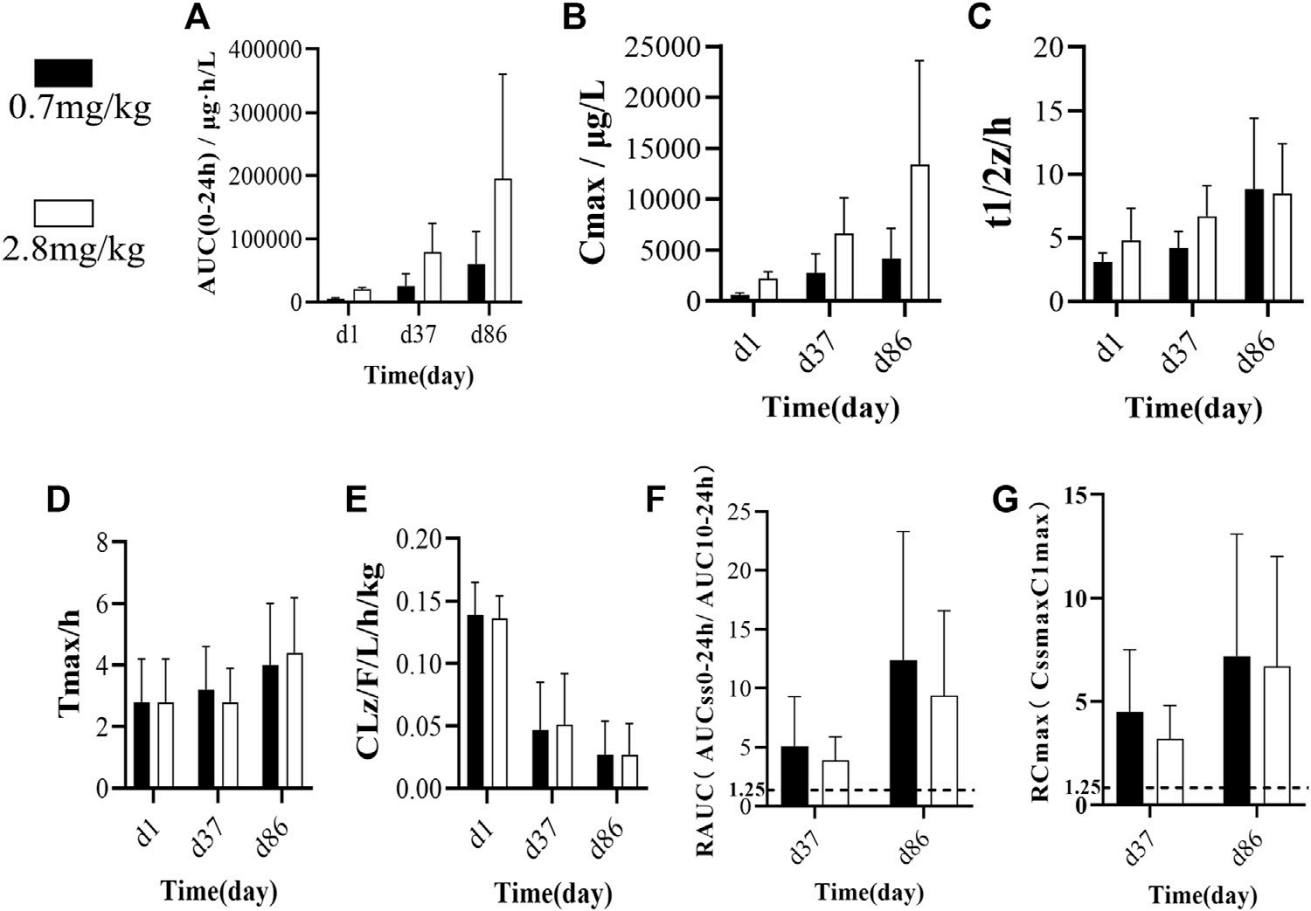
**(A)** Average AUC(0-24 h) of single/multiple subcutaneous injections of FGF-21 in cynomolgus monkeys at different doses and different times(n = 10). **(B–E)** Comparison of toxicity kinetics parameters **(B)**: Cmax/μg/L, **(C)** t1/2/h, **(D)** Tmax/h, **(E)** CLz/F/L/h/kg) in cynomolgus monkeys after single or multiple subcutaneous injections of different doses of FGF-21 ( $\bar{X}\pm s$ , n = 10). **(F,G)** Comparison of drug accumulation index R in cynomolgus monkeys after single/multiple subcutaneous injections of different doses of FGF-21(F:RAUC, **(G)** RCmax).

We analyzed common toxicokinetic parameters, including AUC_(0-24h)_, C_max_, t_1/2z_, T_max_, and CLz/F. The AUC_(0-24h)_ values (Figure 2A) in the low-dose group on d1, d37, and d86 were 5253, 25268, and 60445 μg h/L, respectively. In the high-dose group, the AUC_(0–24h)_ values on d1, d37, and d86 were 19964, 78999, and 195282 μg h/L, respectively (Tables 2, 3).

**TABLE 2:** Comparison of main toxicokinetic parameters of cynomolgus monkey after s.c. FGF-21 (0.7 mg/kg) at different times ( $\bar{X}\pm s$ ).

Does (0.7 mg/kg, n = 10)		Days of administration		
Parameter	Unit	1	37	86
AUC_(0–24h)_	μg·h/L	5253 ± 1,587	25268 ± 19506	60445 ± 50891
C_max_	μg/L	621.1 ± 174.2	2,767.9 ± 1877.8	4,182.0 ± 2,963.1
t_1/2z_	h	3.1 ± 0.7	4.2 ± 1.3	8.8 ± 5.6
T_max_	h	2.8 ± 1.4	3.2 ± 1.4	4.0 ± 2.0
CLz/F	L/h/kg	0.139 ± 0.026	0.047 ± 0.038	0.027 ± 0.027

**TABLE 3:** Comparison of main toxicokinetic parameters of cynomolgus monkey after s.c. FGF-21 (2.8 mg/kg) at different times ( $\bar{X}\pm s$ ).

Does (2.8 mg/kg, n = 10)		Days of administration		
Parameter	Unit	1	37	86
AUC_(0–24h)_	μg·h/L	19964 ± 3,185	78999 ± 45790	195282 ± 165291
C_max_	μg/L	2,196.8 ± 683.2	6,637.5 ± 3,500.0	13430.4 ± 10153.6
t_1/2z_	h	4.8 ± 2.5	6.7 ± 2.4	8.5 ± 3.9
T_max_	h	2.8 ± 1.4	2.8 ± 1.1	4.4 ± 1.8
CLz/F	L/h/kg	0.136 ± 0.018	0.051 ± 0.041	0.027 ± 0.025

In the low-dose group, the C_max_ values (Figure 2B) on d1, d37, and d86 were 621.1, 2,767.9, and 4,182.0 μg/L, respectively. In the high-dose group, the C_max_ values on d1, d37, and d86 were 2,196.8, 6637.5, and 13430.4 μg/L, respectively (Tables 2, 3). In the low-dose group, the t_1/2z_ values (Figure 2C) on d1, d37, and d86 were 3.1, 4.2, and 8.8 h, respectively. In the high-dose group, the t_1/2z_ values on d1, d37, and d86 were 4.8, 6.7, and 8.5 h, respectively (Tables 2, 3).

In the low-dose group, the T_max_ values (Figure 2D) on d1, d37, and d86 were 2.8, 3.2, and 4.0 h, respectively. In the high-dose group, the T_max_ values on d1, d37, and d86 were 2.8, 2.8, and 4.4 h, respectively (Tables 2, 3).

In the low-dose group, the CLz/F values (Figure 2E) of serum FGF-21 on d1, d37, and d86 were 0.139, 0.047, and 0.027 L/h/kg, respectively. In the high-dose group, the CLz/F values on d1, d37, and d86 were 0.136, 0.051, and 0.027 L/h/kg, respectively (Tables 2, 3). In addition, we calculated the drug accumulation index R to evaluate the accumulation of FGF-21 in cynomolgus monkeys after subcutaneous administration of low and high doses of FGF-21. The R values are as follows: R-AUC: on d37, 5.1 (L) and 3.9 (H), and d86, 12.4 (L) and 9.4 (H); R-C_max_: on d37, 4.5 (L) and 3.2 (H), and on d86, 7.2 (L) and 6.7 (H) (Figures 2F,G). All R values are higher than the accumulated judgment standard (1.25).

### 3.4 Hematology and blood biochemical index test results

After subcutaneous injection of different doses of FGF-21 in cynomolgus monkeys, hematological tests were performed at different time points during the administration period and recovery period. The results showed that the mean value of prothrombin time (PT) in the high dose group was significantly higher than that in the vehicle control group at the same time points (16.1 ± 6.2) vs. (11.0 ± 1.5), (*p* < 0.05). This effect may be due to factors such as occurrence time (d87), dose (high dose), and reversibility. (Tables 4, 5, 6, 7, 8). Similarly, blood biochemical tests were performed at different times during the administration period and the convalescence period. We observed a higher mean value of aspartate aminotransferase (AST) in the high dose group (92.6 ± 27.1 U/L) than in the vehicle control group (60.9 ± 8.8 U/L) (*p* < 0.01). The AST (160.3U/L) of one animal on d87 was higher than that of the other time points (d0, d30, d65, rd29, data not displayed). Transient elevation of AST (d87) does not rule out the effect of repeated subcutaneous administration (3 months of administration) at high doses on individual animal blood biochemical AST (Tables 9, 10, 11, 12, 13). In addition, the mean values of some hematological and blood biochemical indicators in the high-dose group were significantly different from those of the vehicle control group at the same period of administration and recovery (*p* < 0.05 or *p* < 0.01). However, these changes were within the normal range of fluctuation. Moreover, there was no obvious time-response relationship and dose correlation, thus, the changes can be considered unrelated to the effect of FGF-21.

**TABLE 4:** Hematological Indexes of each group of cynomolgus monkey s.c.FGF-21 before administration(d0) ( $\bar{X}\pm s$ , n = 10).

Index	Vehicle (0 mg/kg)	Low dose 0.7 mg/kg	High dose (2.8 mg/kg)
WBC(×10^9^/L)	10.16 ± 2.47	11.53 ± 2.76	10.10 ± 1.99
RBC(×10^12^/L)	5.37 ± 0.43	5.25 ± 0.32	5.59 ± 0.53
Hb(g/dL)	133 ± 9	130 ± 7	137 ± 8
HCT (%)	43.4 ± 2.7	42.5 ± 2.6	44.3 ± 3.0
MCV (fL)	80.9 ± 2.9	81.0 ± 2.8	79.5 ± 3.9
MCH(Pg)	24.9 ± 0.9	24.8 ± 0.8	24.5 ± 1.3
MCHC(g/dL)	307 ± 7	307 ± 6	309 ± 7
PLT(×10^9^/L)	477 ± 105	451 ± 110	467 ± 112
N (%)	51.9 ± 10.7	47.9 ± 12.1	44.5 ± 11.4
L (%)	40.8 ± 9.1	47.0 ± 12.5	49.7 ± 10.7
M (%)	4.2 ± 1.3	3.0 ± 0.7*	3.6 ± 1.2
E (%)	1.9 ± 2.0	0.7 ± 0.4	0.6 ± 0.5
RET (%)	1.69 ± 0.76	1.59 ± 0.56	1.58 ± 0.26
PT(s)	10.9 ± 1.1	11.0 ± 1.2	12.6 ± 2.9
APTT(s)	28.9 ± 3.6	31.1 ± 6.0	33.6 ± 6.1

*p<0.05 (compared with vehicle control).

**TABLE 5:** Hematological Indexes of each group of cynomolgus monkeys s.c.FGF-21 during the administration period (d30) ( $\bar{X}\pm s$ , n = 10).

Index	Vehicle (0 mg/kg)	Low dose 0.7 mg/kg	High dose (2.8 mg/kg)
WBC(×10^9^/L)	10.98 ± 2.00	10.39 ± 2.72	10.71 ± 2.34
RBC(×10^12^/L)	5.63 ± 0.42	5.27 ± 0.40	5.60 ± 0.34
Hb(g/dL)	137 ± 8	129 ± 10**	133 ± 8
HCT (%)	45.0 ± 2.3	41.9 ± 3.2*	43.9 ± 3.0
MCV (fL)	80.1 ± 4.9	79.4 ± 3.0	78.5 ± 5.6
MCH(Pg)	24.4 ± 1.0	24.4 ± 0.9	23.8 ± 1.1
MCHC(g/dL)	305 ± 11	308 ± 8	304 ± 18
PLT(×10^9^/L)	486 ± 106	435 ± 126	481 ± 145
N (%)	52.2 ± 11.7	47.4 ± 13.3	40.8 ± 17.4
L (%)	40.4 ± 10.6	46.5 ± 12.4	53.0 ± 16.4
M (%)	4.6 ± 1.3	3.8 ± 1.2	3.9 ± 1.5
E (%)	1.3 ± 1.3	0.9 ± 0.9	0.7 ± 0.5
RET (%)	1.21 ± 0.52	1.48 ± 0.71	2.06 ± 0.65**
PT(s)	11.9 ± 2.6	10.3 ± 1.7	14.8 ± 4.2
APTT(s)	31.3 ± 5.8	28.1 ± 4.3	36.1 ± 10.7

*p< 0.05 (compared with vehicle control).

**TABLE 6:** Hematological Indexes of each group of cynomolgus monkeys s.c.FGF-21 during the administration period (d65) ( $\bar{X}\pm s$ , n = 10).

Index	Vehicle (0 mg/kg)	Low dose 0.7 mg/kg	High dose (2.8 mg/kg)
WBC(×10^9^/L)	12.28 ± 3.20	10.61 ± 2.31	14.37 ± 4.95
RBC(×10^12^/L)	5.49 ± 0.39	5.24 ± 0.44	5.58 ± 0.37
Hb(g/dL)	134 ± 7	126 ± 10	130 ± 9
HCT (%)	44.5 ± 2.3	41.9 ± 2.7*	44.3 ± 3.7
MCV (fL)	81.2 ± 3.0	80.2 ± 3.0	79.4 ± 4.4
MCH(Pg)	24.4 ± 1.0	24.0 ± 1.3	23.4 ± 1.2
MCHC(g/dL)	300 ± 10	299 ± 10	295 ± 11
PLT(×10^9^/L)	446 ± 82	430 ± 83	476 ± 139
N (%)	48.6 ± 9.1	39.9 ± 10.4	40.5 ± 9.7
L (%)	42.9 ± 7.2	51.3 ± 9.4*	51.0 ± 8.8*
M (%)	5.1 ± 0.9	3.8 ± 1.2*	4.1 ± 1.4
E (%)	1.5 ± 1.3	2.9 ± 3.2	2.0 ± 1.4
RET (%)	1.45 ± 0.35	1.50 ± 0.54	1.82 ± 0.57
PT(s)	11.6 ± 1.9	11.8 ± 2.6	12.5 ± 1.8
APTT(s)	29.7 ± 3.9	30.9 ± 5.2	30.0 ± 2.5

*p< 0.05 (compared with vehicle control).

**TABLE 7:** Hematological Indexes of each group of cynomolgus monkeys s.c.FGF-21 during the administration period (d87) ( $\bar{X}\pm s$ , n = 10).

Index	Vehicle (0 mg/kg)	Low dose 0.7 mg/kg	High dose (2.8 mg/kg)
WBC(×10^9^/L)	10.37 ± 2.01	11.66 ± 2.78	10.92 ± 2.78
RBC(×10^12^/L)	5.65 ± 0.41	5.27 ± 0.42	5.37 ± 0.36
Hb(g/dL)	135 ± 9	123 ± 9**	123 ± 7**
HCT (%)	44.6 ± 3.2	40.5 ± 2.6**	40.9 ± 2.3**
MCV (fL)	79.1 ± 3.4	77.1 ± 3.6	76.1 ± 2.9
MCH(Pg)	24.0 ± 1.1	23.4 ± 1.3	23.0 ± 1.0*
MCHC(g/dL)	303 ± 10	303 ± 7	302 ± 9
PLT(×10^9^/L)	451 ± 66	365 ± 63**	404 ± 93
N (%)	43.0 ± 6.4	54.7 ± 8.8**	38.8 ± 11.2
L (%)	49.4 ± 5.2	39.4 ± 7.5**	54.1 ± 11.1
M (%)	4.4 ± 1.0	3.0 ± 1.0**	3.6 ± 1.2
E (%)	1.4 ± 1.9	1.3 ± 1.4	1.6 ± 1.2
RET (%)	1.60 ± 0.43	1.41 ± 0.47	1.35 ± 0.29
PT(s)	11.0 ± 1.5	11.8 ± 3.6	16.1 ± 6.2*
APTT(s)	26.8 ± 2.7	26.9 ± 4.6	34.3 ± 11.2

*p< 0.05 (compared with vehicle control).

**TABLE 8:** Hematological Indexes of each group of cynomolgus monkeys s.c.FGF-21 during the administration period (rd29) ( $\bar{X}\pm s$ , n = 4).

Index	Vehicle (0 mg/kg)	Low dose 0.7 mg/kg	High dose (2.8 mg/kg)
WBC(×10^9^/L)	13.18 ± 4.45	11.50 ± 2.41	13.16 ± 2.13
RBC(×10^12^/L)	5.83 ± 0.33	5.82 ± 0.47	5.64 ± 0.14
Hb(g/dL)	141 ± 9	137 ± 14	135 ± 9
HCT (%)	45.4 ± 2.5	45.0 ± 4.6	44.6 ± 3.5
MCV (fL)	77.8 ± 3.8	77.3 ± 3.0	79.1 ± 5.6
MCH(Pg)	24.2 ± 1.8	23.5 ± 0.8	24.0 ± 1.4
MCHC(g/dL)	310 ± 22	304 ± 6	304 ± 4
PLT(×10^9^/L)	392 ± 43	421 ± 94	392 ± 45
N (%)	36.6 ± 10.2	31.9 ± 5.4	29.0 ± 11.4
L (%)	55.6 ± 8.6	60.4 ± 4.2	63.2 ± 9.8
M (%)	3.5 ± 0.4	3.9 ± 1.3	4.3 ± 1.5
E (%)	1.5 ± 1.1	1.1 ± 0.6	0.7 ± 0.6
RET (%)	1.70 ± 0.30	1.65 ± 0.38	1.86 ± 0.54
PT(s)	11.7 ± 1.2	11.1 ± 1.1	12.6 ± 2.8
APTT(s)	30.8 ± 5.8	30.2 ± 5.7	30.4 ± 5.0

*p< 0.05 (compared with vehicle control).

**TABLE 9:** Before administration of cynomolgus monkey s.c.FGF-21 (d0), blood biochemical Indexes of animals in each group ( $\bar{X}\pm s$ , n = 10).

Index	Vehicle (0 mg/kg)	Low dose 0.7 mg/kg	High dose (2.8 mg/kg)
ALT(U/L)	54.2 ± 26.8	57.5 ± 26.7	63.9 ± 27.0
AST(U/L)	72.5 ± 17.6	67.9 ± 10.3	61.9 ± 8.6
ALP(U/L)	319.9 ± 78.1	326.6 ± 92.9	373.5 ± 100.2
BUN (mmol/L)	6.30 ± 0.82	7.36 ± 1.61	8.04 ± 3.12
Crea (umol/L)	90.0 ± 10.0	100.4 ± 10.0*	97.5 ± 12.4
TP(g/L)	83.9 ± 6.3	85.6 ± 6.6	84.7 ± 4.3
ALB(g/L)	41.0 ± 3.7	40.3 ± 3.4	40.8 ± 2.5
GLU (mmol/L)	4.54 ± 0.91	4.37 ± 1.03	4.58 ± 0.89
TCHO (mmol/L)	2.63 ± 0.71	2.55 ± 0.77	2.60 ± 0.68
TBIL (umol/L)	4.02 ± 0.64	4.02 ± 1.43	3.20 ± 0.74*
TG (mmol/L)	0.24 ± 0.10	0.25 ± 0.08	0.31 ± 0.13
CK(U/L)	240.7 ± 80.3	393.9 ± 372.8	248.3 ± 91.5
γ-GT (U/L)	65.1 ± 43.5	45.8 ± 9.0	64.7 ± 30.5
K^+^ (mmol/L)	5.11 ± 0.47	5.46 ± 0.74	5.31 ± 0.92
Na^+^ (mmol/L)	145.5 ± 3.4	145.0 ± 3.8	147.0 ± 3.9
Cl^−^(mmol/L)	104.6 ± 4.9	106.0 ± 4.4	106.5 ± 5.2

*p< 0.05 (compared with vehicle control).

**TABLE 10:** Before administration of cynomolgus monkey s.c.FGF-21 (d30), blood biochemical Indexes of animals in each group ( $\bar{X}\pm s$ , n = 10).

Index	Vehicle (0 mg/kg)	Low dose 0.7 mg/kg	High dose (2.8 mg/kg)
ALT(U/L)	43.9 ± 24.6^a^	49.5 ± 19.3	51.8 ± 26.0
AST(U/L)	77.4 ± 35.8^a^	68.0 ± 13.1	67.3 ± 8.8
ALP(U/L)	277.9 ± 51.6	254.4 ± 78.6	289.0 ± 95.7
BUN (mmol/L)	6.83 ± 1.21	7.91 ± 1.15	7.87 ± 1.34
Crea (umol/L)	92.5 ± 14.8	96.5 ± 9.6	94.4 ± 14.8
TP(g/L)	84.8 ± 4.5	83.9 ± 8.1	82.8 ± 5.2
ALB(g/L)	38.8 ± 4.3	37.5 ± 3.3	36.3 ± 4.1
GLU (mmol/L)	5.72 ± 1.91	5.28 ± 1.01	6.11 ± 2.89
TCHO (mmol/L)	2.63 ± 0.73	2.31 ± 0.59	2.51 ± 0.88
TBIL (umol/L)	3.56 ± 0.65	4.39 ± 1.35	3.33 ± 0.59
TG (mmol/L)	0.44 ± 0.25	0.24 ± 0.07*	0.29 ± 0.10
CK(U/L)	251.9 ± 89.2	197.0 ± 85.0	264.8 ± 116.7
γ-GT (U/L)	48.6 ± 14.1	36.6 ± 6.0*	51.8 ± 33.2
K^+^ (mmol/L)	5.55 ± 0.77	5.12 ± 0.64	4.97 ± 0.77
Na^+^ (mmol/L)	149.0 ± 4.4	148.4 ± 3.5	145.7 ± 4.5
Cl^−^(mmol/L)	106.9 ± 5.0	106.2 ± 5.7	104.0 ± 5.9

*p< 0.05 (compared with vehicle control), ^a^:n = 9.

**TABLE 11:** Before administration of cynomolgus monkey s.c.FGF-21 (d65), blood biochemical Indexes of animals in each group ( $\bar{X}\pm s$ , n = 10).

Index	Vehicle (0 mg/kg)	Low dose 0.7 mg/kg	High dose (2.8 mg/kg)
ALT(U/L)	33.5 ± 11.7	42.5 ± 13.4	36.6 ± 12.2
AST(U/L)	66.8 ± 14.7	63.6 ± 7.1	60.1 ± 11.9
ALP(U/L)	284.1 ± 81.1	261.6 ± 70.9	308.4 ± 138.0
BUN (mmol/L)	6.60 ± 1.32	7.72 ± 1.89	7.72 ± 1.71
Crea (umol/L)	92.8 ± 9.9	92.5 ± 6.2	93.7 ± 11.6
TP(g/L)	76.5 ± 7.1	80.8 ± 6.1	80.3 ± 4.0
ALB(g/L)	37.7 ± 2.1	36.7 ± 2.5	35.5 ± 3.5
GLU (mmol/L)	4.57 ± 0.54	5.00 ± 0.99	5.86 ± 1.45*
TCHO (mmol/L)	2.88 ± 0.74	2.75 ± 0.77	2.94 ± 0.79
TBIL (umol/L)	3.25 ± 0.73	3.54 ± 0.79	2.93 ± 0.94
TG (mmol/L)	0.30 ± 0.11	0.26 ± 0.06	0.37 ± 0.13
CK(U/L)	374.3 ± 268.4	214.9 ± 76.2	219.2 ± 144.4
γ-GT (U/L)	52.4 ± 14.9	37.0 ± 7.2*	52.8 ± 31.0
K^+^ (mmol/L)	4.99 ± 0.29	4.90 ± 0.48	4.86 ± 0.32
Na^+^ (mmol/L)	149.0 ± 2.7	150.1 ± 2.6	148.7 ± 2.0
Cl^−^(mmol/L)	116.1 ± 2.4	117.3 ± 2.2	116.3 ± 3.5

*p< 0.05 (compared with vehicle control).

**TABLE 12:** Before administration of cynomolgus monkey s.c.FGF-21 (d87), blood biochemical Indexes of animals in each group ( $\bar{X}\pm s$ , n = 10).

Index	Vehicle (0 mg/kg)	Low dose 0.7 mg/kg	High dose (2.8 mg/kg)
ALT(U/L)	35.0 ± 14.2	46.4 ± 16.6	50.8 ± 18.2*
AST(U/L)	60.9 ± 8.8	84.5 ± 32.5	92.6 ± 27.1**
ALP(U/L)	333.8 ± 94.0	272.0 ± 73.4	310.5 ± 111.6
BUN (mmol/L)	5.71 ± 1.11	7.05 ± 0.98*	6.43 ± 1.16
Crea (umol/L)	88.1 ± 6.7	88.6 ± 8.2	90.4 ± 9.7
TP(g/L)	79.2 ± 5.3	77.6 ± 5.3	77.0 ± 4.5
ALB(g/L)	39.7 ± 1.5	36.9 ± 3.2*	35.9 ± 1.9**
GLU (mmol/L)	4.31 ± 1.09	4.50 ± 0.92	5.32 ± 1.26
TCHO (mmol/L)	3.29 ± 0.71	2.93 ± 0.68	2.97 ± 0.69
TBIL (umol/L)	4.47 ± 1.17	5.59 ± 1.71	4.47 ± 1.37
TG (mmol/L)	0.28 ± 0.09	0.31 ± 0.08	0.37 ± 0.10*
CK(U/L)	609.6 ± 762.8	576.8 ± 635.8	572.4 ± 551.8
γ-GT (U/L)	58.2 ± 16.3	37.0 ± 5.7**	54.2 ± 26.2
K^+^ (mmol/L)	5.40 ± 0.61	4.83 ± 0.47*	4.91 ± 0.40*
Na^+^ (mmol/L)	147.0 ± 2.1	145.3 ± 2.3	145.6 ± 3.4
Cl^−^(mmol/L)	112.9 ± 1.5	111.7 ± 1.6	112.4 ± 3.1

*p< 0.05, **: *p* < 0.01 (compared with vehicle control).

**TABLE 13:** Before administration of cynomolgus monkey s.c.FGF-21 (rd29), blood biochemical Indexes of animals in each group ( $\bar{X}\pm s$ , n = 4).

Index	Vehicle (0 mg/kg)	Low dose 0.7 mg/kg	High dose (2.8 mg/kg)
ALT(U/L)	29.2 ± 12.7	34.9 ± 7.6	33.8 ± 11.6
AST(U/L)	58.0 ± 17.0	55.9 ± 7.8	69.3 ± 42.1
ALP(U/L)	400.4 ± 143.3	305.9 ± 29.3	317.3 ± 112.8
BUN (mmol/L)	5.63 ± 0.39	7.16 ± 0.74*	6.72 ± 1.94
Crea (umol/L)	87.3 ± 7.4	90.8 ± 9.4	82.3 ± 5.3
TP(g/L)	73.6 ± 3.5	79.3 ± 5.0	74.9 ± 2.2
ALB(g/L)	37.4 ± 1.2	38.5 ± 4.9	37.8 ± 2.6
GLU (mmol/L)	4.17 ± 1.04	5.19 ± 1.20	4.77 ± 0.96
TCHO (mmol/L)	3.12 ± 0.67	3.60 ± 1.32	3.10 ± 0.66
TBIL (umol/L)	2.71 ± 0.42	2.83 ± 0.89	3.18 ± 2.16
TG (mmol/L)	0.38 ± 0.14	0.31 ± 0.11	0.44 ± 0.12
CK(U/L)	425.8 ± 547.9	221.6 ± 111.6	410.6 ± 452.2
γ-GT (U/L)	66.3 ± 19.1	45.9 ± 16.5	82.0 ± 54.6
K^+^ (mmol/L)	4.26 ± 0.36	4.22 ± 0.25	4.42 ± 0.67
Na^+^ (mmol/L)	144.3 ± 2.5	143.4 ± 0.8	145.0 ± 5.3
Cl^−^(mmol/L)	104.4 ± 6.7	103.4 ± 6.4	104.8 ± 9.4

*p< 0.05 (compared with vehicle control).

### 3.5 General observation and histopathology

**FIGURE 3:**
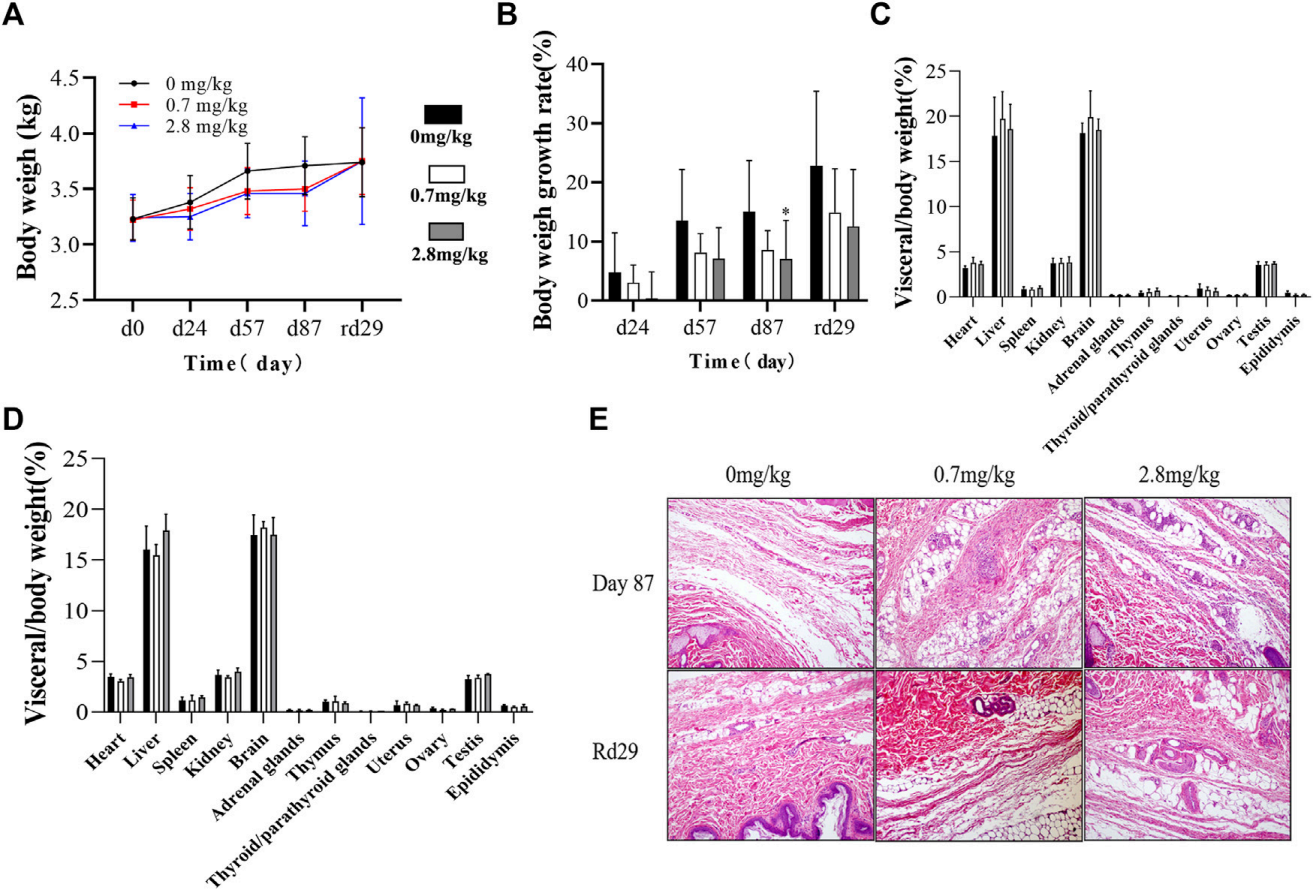
Effects of different doses of FGF-21 subcutaneously injected on body weight, organs and histopathological analysis of cynomolgus monkeys. **(A,B)** Average body weight **(A)** and growth rate **(B)** of cynomolgus monkeys during the administration and recovery periods of FGF-21( $\bar{X}\pm s$ , n = 10). **(C)** Average organ coefficient of cynomolgus monkeys at the end of administration period (D87) after subcutaneous injection of FGF-21( $\bar{X}\pm s$ , n = 6). **(D)** Average organ coefficient of cynomolgus monkeys injected subcutaneously with FGF-21 at the end of recovery period (rd29) ( $\bar{X}\pm s$ , n = 4). **(E)** HE staining of paraffin sections of cynomolgus monkey skin from subcutaneous injection area (The black arrows indicate areas of inflammatory cells infiltration and the red arrows indicate areas of tissue necrosis).

During the administration and recovery, there were no abnormalities in the animals’ appearance, physical signs, activity, behavior, gait, and breathing. Abnormal secretions from the eyes and nose, loose stools, soft stools, skin erythema, and skin edema were not observed throughout the study period. However, compared to the control group, animals in the FGF-21 groups, especially the high-dose group, had reduced body weight and a slower rate of weight gain (Figures 3A,B).

Necropsy results showed that there were no pathological changes in the shape, color, location, and texture of the organs. The cardiac organ coefficient in each FGF-21 group was slightly higher than that of the control group (*p* < 0.05). However, no significant relationship between drug dose and heart weight was observed (*p* > 0.05). No significant difference was found in other tissue weights and organ coefficients (Figures 3C,D).

We examined the histopathology of monkey organs. The results showed that repeated subcutaneous injection of FGF-21 only mildly stimulated the subcutaneous injection site (inflammatory cell infiltration and mild tissue necrosis). After 1 month of recovery, the skins of the animals appeared normal (Figure 3E).

## 4 Discussion

Toxicokinetics is the study of the kinetics of absorption, distribution, metabolism, and excretion of different drug dosages under the conditions of long-term toxicity evaluation (Dixit et al., 2003). Toxicokinetics studies are utilized to understand the relationship between drug dose and toxicity. Furthermore, they are important in clinical research as they are used in determining the plasma limits and the calculation of safety margins (Baldrick, 2003).

FGF-21 is a metabolic hormone that is predominantly produced by the liver (BonDurant and Potthoff, 2018). It can promote the homeostasis of metabolism in disease and health, especially in models of obesity and diabetes (Adams et al., 2013; Sanyal et al., 2019; Harrison et al., 2021). However, the administration cycle of FGF-21 for metabolic diseases is often long. FGF-21 accumulation during the treatment process and the potential toxicity have been difficult to quantify. As such, there have been little to no reports on the toxicokinetics of FGF-21. The characteristics of FGF-21 accumulation and toxicity have also been poorly elucidated. In the present study, a validated ELISA kit was used to study the toxicokinetics of repeated treatment of FGF-21 in cynomolgus monkeys for 3 months.

Administration of FGF-21 to cynomolgus monkeys for 3 months, FGF-21 levels in serum increased with the increase of the dose and administration times. The correlation between AUC and dose indicates that FGF-21 tends to accumulate following repeated treatment. The toxicokinetic results showed that the average AUC of FGF-21 in the low- and high-dose groups gradually increased over time. The average AUC value in the low- and high-dose groups on d37 was 3–5 times higher than that on d1 (low-dose group: 25268 vs. 5253 μg h/L; high-dose group: 78999 vs. 19964 μg h/L). The average AUC in the low- and high-dose groups on d86 was 5–12 times higher than that on d1 (low-dose group: 60445 vs. 5253 μg h/L; high-dose group: 195282 vs. 19964 μg h/L).

Similarly, the Cmax value in the low- and high-dose groups was 3–5 times higher on d37 than on d1 (low-dose group: 2,767.9 vs. 621.1 μg/L; high-dose group: 6637.5 vs. 2,196.8 μg/L). On d86, the Cmax value was 3–7 times higher than on d1 (low-dose group: 4,182.0 vs. 621.1 μg/L; high-dose group: 13430.4 vs. 2,196.8 μg/L). The AUC and Cmax values in the low- and high-dose groups had increased approximately 2-3-fold by the end of the administration of FGF-21 (d86) compared to d37. On the same day of administration, the differences in t1/2z, Tmax, and CLz/F between the low- and high-dose groups were not significant. However, over time, the t1/2z of each group was prolonged (low-dose group: 3.1 h on d1, 4.2 h on d37, and 8.8 h on d86; high-dose group: 4.8 h on d1, 6.7 h on d37, and 8.5 h on d86). The CLz/F value decreased over time. While the range of Tmax was within 2.4–4.4 h, it was slightly delayed over time. These findings indicate that serum FGF-21 levels increase over time and that the degree of accumulation is influenced by individual differences that impact the rate of absorption.

The accumulation index R is used to quantify the degree of drug accumulation. The calculated cumulative index R (R-AUC: on d37, 5.1 in the low-dose group and 3.9 in the high-dose group; on d86, 12.4 in the low-dose group and 9.4 in the high-dose group) and R-Cmax (on d37, 4.5 in the low-dose group and 3.2 in the high-dose group; on d86, 7.2 in the low-dose group and 6.7 in the high-dose group) showed that long-term skin injection of FGF-21 leads to accumulation in cynomolgus monkeys (accumulation index 95% CIL >1.25, i.e., there is a tendency to accumulate).

Hematological tests are performed to diagnose indicators of blood cell abnormalities, infection, anemia, and hematopoietic dysfunction. Blood biochemical tests are used to evaluate viscera function (liver, kidney, etc.) and diagnose some diseases. To fully assess the effects of FGF-21 on cynomolgus monkeys, blood was collected from cynomolgus monkeys at various points during administration. The blood was used for hematology and blood biochemical tests. Results showed that high doses of FGF-21 significantly alter PT and AST, and this effect can be restored after discontinuation. Examining individual data of 10 cynomolgus monkeys in high dose group on day 87, not all cynomolgus monkeys had elevated PT and AST. Three cynomolgus monkeys with elevated PT (3/10); One cynomolgus monkey had an elevated AST (1/10). Changes in PT and AST during FGF-21 therapy may require attention in clinical settings. FGF-21 had no significant effect on other hematological and blood biochemical indexes. These indicators are mainly used to indicate whether the body has inflammation, infection, liver and kidney damage, anemia, hematopoietic dysfunction, abnormal blood sugar and electrolyte homeostasis. Therefore, Therefore, our results suggest that continued administration of FGF-21 does not affect blood, electrolyte homeostasis, liver and kidney function, nor does it cause disease in cynomolgus monkeys. In addition, a pathological examination of the liver and kidney did not reveal any lesions. Therefore, it is likely that the accumulation of FGF-21 in cynomolgus monkeys does not induce liver and kidney injury and/or reduce liver and kidney size and weight.

In this study, we also found that animals in the FGF-21 group, especially the high-dose group, lost weight and had a slower rate of weight gain compared to those in the control group (Figures 3A,B). FGF-21 appears to have a greater effect on body weight in male cynomolgus monkeys compared to females (Supplementary Figure S1). This phenomenon may be due to the different effects of FGF-21 on the metabolism of different sex (Makarova et al., 2021a; Makarova et al., 2021b), however, further studies are needed to confirm this. No difference in the parameters of toxicity kinetics and organ coefficients due to gender difference was observed (Supplementary Tables S1, S2; Supplementary Figure S2). Some studies have demonstrated that FGF-21 enhances energy expenditure by inducing brown fat activity, thereby concomitantly leading to weight loss (Emanuelli et al., 2015; BonDurant et al., 2017). According to our observations, there were no significant changes in the appetite and food intake of cynomolgus monkeys during the administration and recovery periods. Compared with body weight, our data showed no significant change in the wet weight of organs in cynomolgus monkeys ((Supplementary Figures S1, S3). Therefore, changes in body weight may be responsible for the observed differences in organ coefficients. The increased organ coefficient decreased after a 1-month recovery period. This may be because during the recovery period, the cynomolgus monkeys did not receive FGF-21 injections, and their body weight returned to normal, resulting in the recovery of their organ coefficients. Therefore, in this study, the changes in body weight of cynomolgus monkeys were associated with higher organ coefficients, rather than FGF-21 directly changing organ wet weight of cynomolgus monkeys. In addition, studies have shown that FGF-21 can regulate bile acid metabolism and may cause gastrointestinal reactions such as diarrhea (Zhang et al., 2017; Chen et al., 2018). While we did not quantify changes in the bile acid profiles of cynomolgus monkeys, we did not observe signs of yellow urine, loose stools, or soft stools throughout the study period.

In conclusion, the serum concentration of FGF-21 in cynomolgus monkeys after subcutaneous injection was determined by ELISA, and the toxicokinetic profile of subcutaneous injections of FGF-21 was analyzed. Although FGF-21 tends to accumulate after repeated subcutaneous injection, this has no significant impact on the clinical signs, weight, and organ functions of cynomolgus monkeys, which indicates that subcutaneously injected FGF-21 is a relatively safe drug in cynomolgus monkeys. Attention should be paid to drug accumulation during clinical use. It is suggested to conduct preliminary experiments or take certain preventive measures, for example, to select the appropriate method of administration. The administration interval must be strictly controlled, and during treatment, individual differences require special attention.

## Data Availability

The raw data supporting the conclusions of this article will be made available by the authors, without undue reservation.
