# Supplementary material for: Toxicokinetics of recombinant human fibroblast growth factor 21 for injection in cynomolgus monkey for 3 months
Source: Front Pharmacol. 2023 May 23;14:1176136. doi: 10.3389/fphar.2023.1176136 (PMC10242211; doi:10.3389/fphar.2023.1176136)
Supplement: Supplementary file 1 [file Table1.docx]

| **Days** | **Parameters** | **Femle (0.7mg/kg, n=5)** | | | | | **Male (0.7mg/kg, n=5)** | | | | | **Significantly different (F vs M, P < 0.05)?** |
| --- | --- | --- | --- | --- | --- | --- | --- | --- | --- | --- | --- | --- |
| **d1** | AUC_(0-24h)_ /μg·h/L | 4791 | 5008 | 4613 | 4791 | 4469 | 4164 | 5024 | 4618 | 9670 | 5383 | no |
|  | C_max_ / μg/L | 2460.3 | 1190.1 | 2984.9 | 1006.6 | 2065.7 | 1867.5 | 2129.6 | 2645.8 | 2801.8 | 2815.5 | no |
|  | t_1/2z_/h | 2.6 | 3.7 | 4.1 | 4 | 2.6 | 2.3 | 2.7 | 2.8 | 2.9 | 3.3 | no |
|  | T_max_/h | 1.5 | 1.5 | 5 | 1.5 | 5 | 3 | 3 | 3 | 1.5 | 3 | no |
|  | CL_z_/F/ L/h/kg | 0.146 | 0.138 | 0.148 | 0.144 | 0.156 | 0.168 | 0.139 | 0.151 | 0.072 | 0.128 | no |
|  |  |  |  |  |  |  |  |  |  |  |  |  |
| **d37** | AUC_(0-24h)_ /μg·h/L | 13905 | 5369 | 15543 | 66957 | 12545 | 39337 | 44202 | 19612 | 27581 | 7628 | no |
|  | C_max_ / μg/L | 1916.7 | 885.1 | 1141.7 | 5793.4 | 1591.6 | 4065.8 | 5770.8 | 2568.5 | 3040.1 | 905.5 | no |
|  | t_1/2z_/h | 2.9 | 2.6 | 4.3 | 4.9 | 2.9 | 4.8 | 6.7 | 5.6 | 3.2 | 4 | no |
|  | T_max_/h | 3 | 3 | 5 | 5 | 3 | 3 | 1.5 | 1.5 | 5 | 1.5 | no |
|  | CL_z_/F/ L/h/kg | 0.05 | 0.13 | 0.044 | 0.01 | 0.055 | 0.017 | 0.015 | 0.034 | 0.025 | 0.09 | no |
|  |  |  |  |  |  |  |  |  |  |  |  |  |
| **d86** | AUC_(0-24h)_ /μg·h/L | 19928 | 9397 | 122074 | 123360 | 12441 | 54399 | 136507 | 75889 | 40379 | 10073 | no |
|  | C_max_ / μg/L | 1887.8 | 1142.1 | 7711.3 | 8029.3 | 1449.8 | 3853.2 | 8397.7 | 4892.4 | 3602.2 | 854.4 | no |
|  | t_1/2z_/h | 5.2 | 5.7 | 21.6 | 14.7 | 5.5 | 6 | 11.7 | 7.7 | 6 | 3.6 | no |
|  | T_max_/h | 3 | 5 | 1.5 | 5 | 1.5 | 3 | 5 | 8 | 3 | 5 | no |
|  | CL_z_/F/ L/h/kg | 0.033 | 0.071 | 0.003 | 0.004 | 0.054 | 0.012 | 0.004 | 0.008 | 0.016 | 0.068 | no |

Table S1. Comparison of main toxicokinetic parameters of different gender's cynomolgus monkey S.C. FGF-21 (0.7mg/kg) at different time points

Table S2. Comparison of main toxicokinetic parameters of different gender's cynomolgus monkey S.C. FGF-21 (2.8mg/kg) at different time points

| **Days** | **Parameters** | **Femle (2.8mg/kg, n=5)** | | | | | **Male (2.8mg/kg, n=5)** | | | | | **Significantly different (F vs M, P < 0.05)?** |
| --- | --- | --- | --- | --- | --- | --- | --- | --- | --- | --- | --- | --- |
| **d1** | AUC_(0-24h)_ /μg·h/L | 18754 | 15788 | 19690 | 14642 | 25483 | 20019 | 19672 | 21261 | 23188 | 21140 | no |
|  | C_max_ / μg/L | 2460.3 | 1190.1 | 2984.9 | 1006.6 | 2065.7 | 1867.5 | 2129.6 | 2645.8 | 2801.8 | 2815.5 | no |
|  | t_1/2z_/h | 3.5 | 8.3 | 3.5 | 9.3 | 7.1 | 3.9 | 3.1 | 2.5 | 3.8 | 2.8 | yes, p=0.04 |
|  | T_max_/h | 3 | 1.5 | 1.5 | 1.5 | 5 | 5 | 3 | 3 | 1.5 | 3 | no |
|  | CL_z_/F/ L/h/kg | 0.148 | 0.151 | 0.141 | 0.16 | 0.097 | 0.137 | 0.141 | 0.131 | 0.119 | 0.132 | no |
|  |  |  |  |  |  |  |  |  |  |  |  |  |
| **d37** | AUC_(0-24h)_ /μg·h/L | 95383 | 37757 | 18949 | 59169 | 124695 | 30891 | 137169 | 116542 | 127382 | 42050 | no |
|  | C_max_ / μg/L | 11346.6 | 2622.5 | 2477.5 | 4942.6 | 10543.6 | 2611.3 | 9981.9 | 7642.2 | 9062.5 | 5144.2 | no |
|  | t_1/2z_/h | 7.2 | 9.9 | 4 | 5.3 | 9.7 | 3.3 | 6.9 | 8.4 | 8 | 4.3 | no |
|  | T_max_/h | 3 | 1.5 | 1.5 | 3 | 1.5 | 5 | 3 | 3 | 3 | 3 | yes, p=0.04 |
|  | CL_z_/F/ L/h/kg | 0.027 | 0.061 | 0.145 | 0.045 | 0.018 | 0.09 | 0.018 | 0.021 | 0.019 | 0.065 | no |
|  |  |  |  |  |  |  |  |  |  |  |  |  |
| **d86** | AUC_(0-24h)_ /μg·h/L | 35266 | 311966 | 44751 | 69364 | 537836 | 78916 | 252371 | 192231 | 343937 | 86182 | no |
|  | C_max_ / μg/L | 3231.6 | 16832 | 3876.9 | 4903 | 33831.6 | 6112.6 | 22017 | 14849.3 | 21437.4 | 7212.2 | no |
|  | t_1/2z_/h | 4.7 | 15.9 | 4.9 | 12 | 9.5 | 5.5 | 6.9 | 7.3 | 12.8 | 5.7 | no |
|  | T_max_/h | 5 | 5 | 5 | 3 | 8 | 5 | 3 | 3 | 1.5 | 5 | no |
|  | CL_z_/F/ L/h/kg | 0.076 | 0.006 | 0.06 | 0.029 | 0.004 | 0.033 | 0.01 | 0.013 | 0.006 | 0.03 | no |
