# Supplementary material for: Toxicokinetics of recombinant human fibroblast growth factor 21 for injection in cynomolgus monkey for 3 months
Source: Front Pharmacol. 2023 May 23;14:1176136. doi: 10.3389/fphar.2023.1176136 (PMC10242211; doi:10.3389/fphar.2023.1176136)
Supplement: Supplementary file 2 [file Presentation1.pdf]

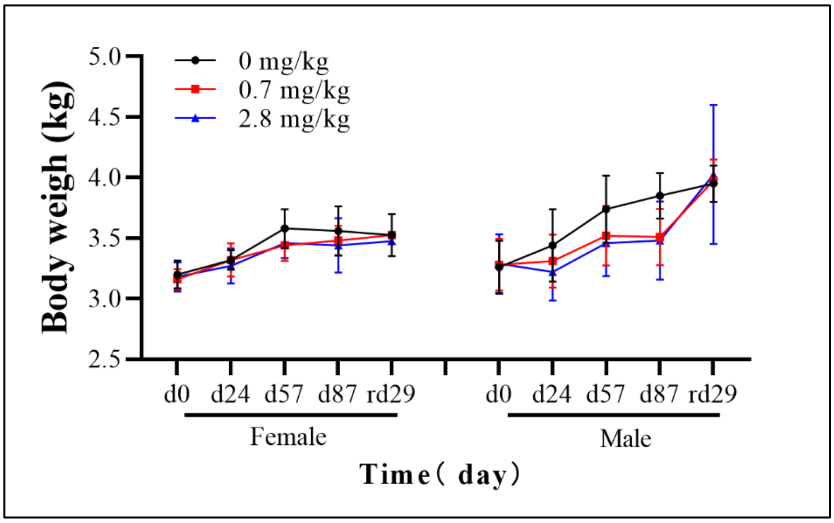

Figure S1. The body weight changes of cynomolgus monkeys of different genders after subcutaneous injection of different doses of FGF-21 at different time points (n=10).

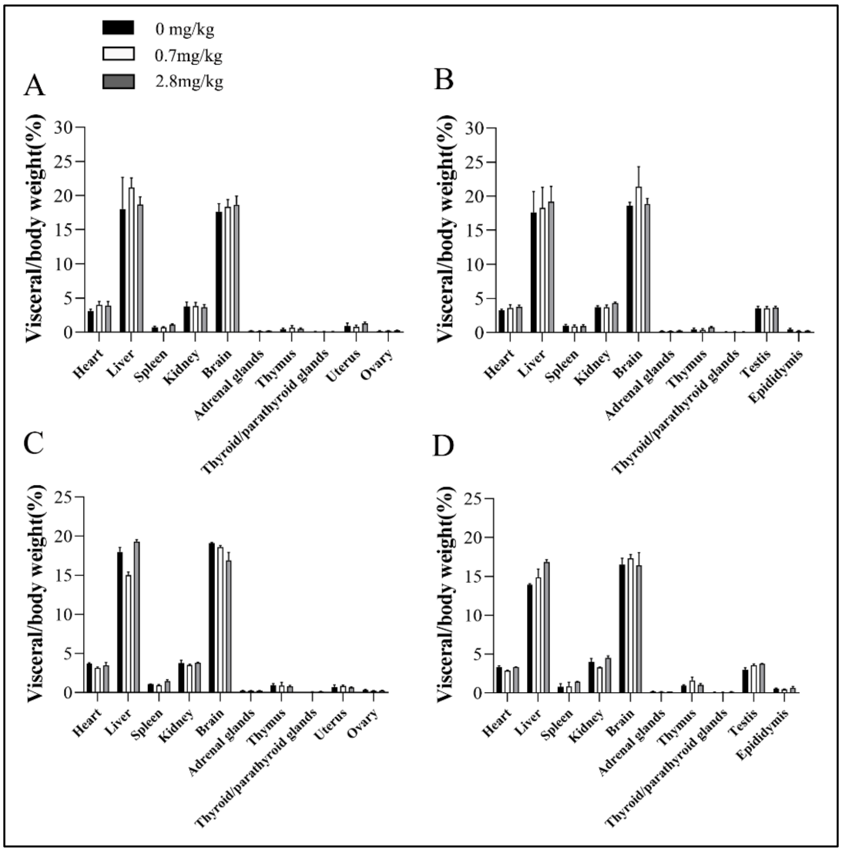

Figure S2. Comparison of organ coefficients after subcutaneous injection of different doses of FGF-21 into cynomolgus monkeys of different genders during the administration and recovery periods. (A) Changes of organ coefficient of female cynomolgus monkeys after FGF-21 administration period (d87, g/kg, n=3). (B) Changes of organ coefficient of male cynomolgus monkeys after FGF-21 administration period (d87, g/kg, n=3). (C) Changes of organ coefficient of female cynomolgus monkeys after recovery periods (rd29, g/kg, n=2). (D) Changes of organ coefficient of male cynomolgus monkeys after recovery periods (rd29, g/kg, n=2).

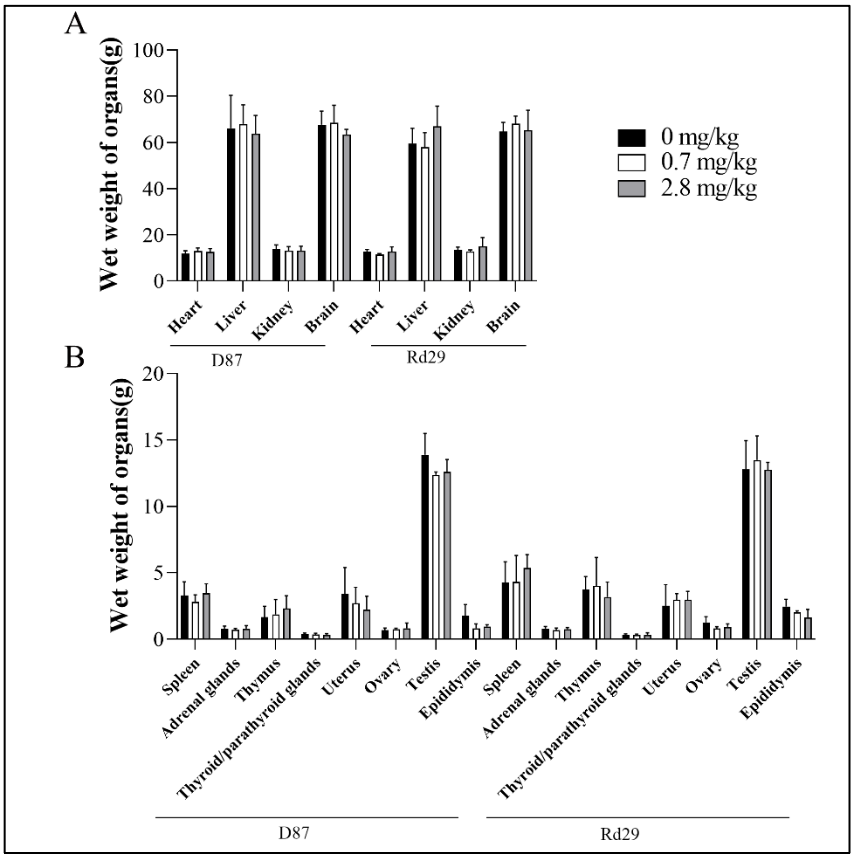

Figure S3. (A) Changes in organ wet weight of individual animals after administration and recovery of s.c.FGF-21 in cynomolgus monkeys -1 (D87: n=6, Rd29: n=4). (B) Changes in organ wet weight of individual animals after administration and recovery of s.c.FGF-21 in cynomolgus monkeys -2 (D87: n=6, Rd29: n=4).
